# Correlation Between Different Parameters of Acute Myocardial Infarction and Obesity

**DOI:** 10.7759/cureus.28572

**Published:** 2022-08-30

**Authors:** Saad A Alqahtani, Attafah M Omeish, Enas M Ghulam, Wael M Alsalim, Hatan J Momenkhan, Olga Vriz, Abdulhalim J Kinsara

**Affiliations:** 1 Cardiology, Prince Khalid Bin Sultan Cardiac Center, Khamis Mushait, SAU; 2 Cardiology, Prince Muhammad Bin Nasser Hospital, Jazan, SAU; 3 Basic Sciences Department, College of Science and Health Professions, King Saud Bin Abdulaziz University for Health Sciences, Jeddah, SAU; 4 Biostatistics, King Abdullah International Medical Research Center, Jeddah, SAU; 5 Academic Affairs, King Salman Armed Forces Hospital, Tabuk, SAU; 6 Family Medicine, King Abdulaziz University, Jeddah, SAU; 7 Cardiology, King Faisal Specialist Hospital and Research Centre, Alfaisal University, Riyadh, SAU; 8 Cardiology, Ministry of National Guard-Health Affairs, King Saud Bin Abdulaziz University for Health Sciences, College of Medicine-Western Region, King Abdullah International Medical Research Center, Jeddah, SAU

**Keywords:** obese, left ventricle mass, body mass index, myocardial infarction, troponin

## Abstract

Objective: To study if obesity had a detrimental effect on troponin after acute coronary syndrome. We investigated the effects of the body mass index (BMI) in post-myocardial infarction (MI) patients, and see the difference in troponin levels and other parameters between normal and overweight patients.

Methodology: A retrospective cohort study was conducted. Data were extracted from the electronic medical files of patients hospitalized due to acute ST-elevation MI to examine the association between BMI and MI. Sixty-one patients were categorized into normal BMI category, overweight, and obese/morbid obesity groups using the baseline measurements, to assess the independent factors associated with a patient with a high BMI who had a MI.

Results: In total, 61 post-myocardial infarction patients with a mean age of 56.9 ± 11.2 years were included in the study. The average BMI was 28.5 ± 6.5 kg/m^2^. Just more than a third (37.4%, n=23) were in the normal BMI category, 19 (31.2%) overweight, and 19 (31.2%) obese/morbid obesity. The mean left ventricle mass was 93.74 ± 32.69 gram and the mean left ventricular ejection fraction was 44.02% ± 10.02. A significant difference in the mean level of troponin and mean heart rate between the body mass index groups (normal vs. overweight groups) was noted. A fair correlation was noted between BMI and left ventricle mass. No statistically significant relation could be linked to high BMI with total cholesterol, low-density lipoprotein (LDL), or aspartate transferase/alanine transaminase (AST/ALT) levels.

Conclusions: In this pilot study, the group with a high BMI had a statistically significant lower troponin level and higher mean heart rate. Such data need to be considered when assessing a patient’s risk. In addition, obese persons with a MI had a higher left ventricle mass.

## Introduction

Over the past three decades, globally the prevalence of overweight and obesity has increased substantially. Saudi Arabia is one of the nations with the highest obesity and overweight prevalence rates, resulting in major consequences in terms of cardiovascular disease (CVD), diabetes, cancer, and ischemic heart disease [1]. Obesity is an independent risk factor for cardiovascular disease and contributes to the development and progression of several other risk factors. It contributes to the risk of coronary heart disease (CHD), either alone or as a part of metabolic syndrome in conjunction with lipid abnormality, especially a high serum low-density lipoprotein (LDL)-cholesterol concentration, hypertension, tobacco smoking, abdominal obesity, diabetes mellitus, physical inactivity, and male gender [2]. 

In the general population, obesity and, especially, morbid obesity are consistently and strongly related to a higher risk of CVD incidence and mortality [3]. Obesity is a complex disease that adversely causes changes in the heart structure and function, both of which might lead to the occurrence of heart failure and the development of CHD, and is associated with reduced overall survival [4]. The changes in the structure and function of the myocardium increase the risk of atrial fibrillation and the occurrence of sudden cardiac death. However, obesity also has a protective effect on the clinical outcome of the underlying CVD, a phenomenon called the obesity paradox [4]. Numerous studies showed the obesity paradox in which overweight and obese people with CVD, including hypertension, heart failure, CHD, and peripheral arterial disease, have a better prognosis compared with non-overweight/non-obese patients [5]. In the group with higher BMI, There was a stronger risk relationship with the incidence of hs-tropnin T level. It was also noted that the association between serum high-sensitivity cardiac troponin T and acute myocardial infarction (MI) in patients with the suspected chronic coronary syndrome is modified by the BMI [6].

The aim of our study was to evaluate the effect of overweight/obesity in patients who sustained an acute myocardial infarction. This article was previously presented as a meeting abstract at the Association of Cardiovascular Nursing and Allied Professions (ACNAP) - EuroHeartCare Congress annual scientific meeting 2022, Madrid, Spain, in July 2022.

## Materials and methods

Study design

This is a retrospective cohort study that included adult patients aged 18 years and above, diagnosed with acute myocardial infarction, diagnosed by elevated troponin I, more than 99th centiles, and with the presence of more than 20 minutes of ischemic pain or electrocardiographic ST-segment elevation. Demographic, clinical, and treatment data were collected, and the BMI values were calculated from the admission height and weight. A fasting blood sample was collected for lipid profile, glucose, renal, and liver function. Patients underwent a comprehensive echocardiogram within 24 hours of admission. The recruited patients were multi-centered, referred patients over one year. The study was approved by the Institutional Review Board at King Abdullah International Medical Research Center with an approval number RJ15/007/J.

Statistical analysis

The analyses were a combination of clinical and statistical judgment and included demographic information, risk factors and medical history, clinical presentation, and treatment. The laboratory investigations were added. The results represent the preliminary analysis of the BMI (as binary variables) and MI measurements. The results are expressed as mean ± standard deviation for the continuous variables. For analyses that modeled BMI as an ordinal variable, we used criteria from the Centers for Disease Control and Prevention to define the BMI categories: normal weight-defined as BMI of 18.5 - 24.9 kg/m^2^, overweight (BMI 25 -29.9 kg/m^2^), obese (30 - 34.9 kg/m^2^), and morbidly obese (more than 35 kg/m^2^). Patients were divided into two groups: normal <25 and obese ≥ 25 for the purpose of analysis. Shapiro-Wilk test was used to check the normality distribution of the variables, and a Student’s t or Mann-Whitney U test for the comparison of the continuous variables as appropriate. The categorical variables were analyzed as frequency and percentage and compared with the χ2 test or Fisher’s exact test. To measure the strength of the association between two variables, the Pearson or Spearman correlation was used. The statistical analysis was performed with the Rstudio, Version 1.2.5033, statistic software package.

## Results

The mean age of participants was 56.9 ± 11.2 years. Fifty were male (82%). Thirty-eight (62.2%) were diabetic, and 28 (45.9%) current smokers, with no history of chronic renal disease, no family history of premature coronary artery disease (CAD), or a prior history of MI. The mean BMI was 28.5 ± 6.5 kg/m^2^. Twenty-three (37.7%) were in the normal weight category, 19 (31.1%) in the overweight category, 10 (16.4%) in the obese category, and 9 (14.8%) were morbidly obese. The mean heart rate was 82.9 ± 18.3 beats per minute. Mean systolic and diastolic blood pressure (BP) were 133.4±20.5 and 82.3 ±12.6 mmHg. The echocardiogram data indicated that 26 (42.6%) of the patients had a normal ejection fraction (EF) (>55%), and left ventricular EF (LVEF) was 44.02 % ± 10.02. The average LV mass (left ventricular mass) was 93.7 ± 32.7 grams (60-120).

**Figure 1:**
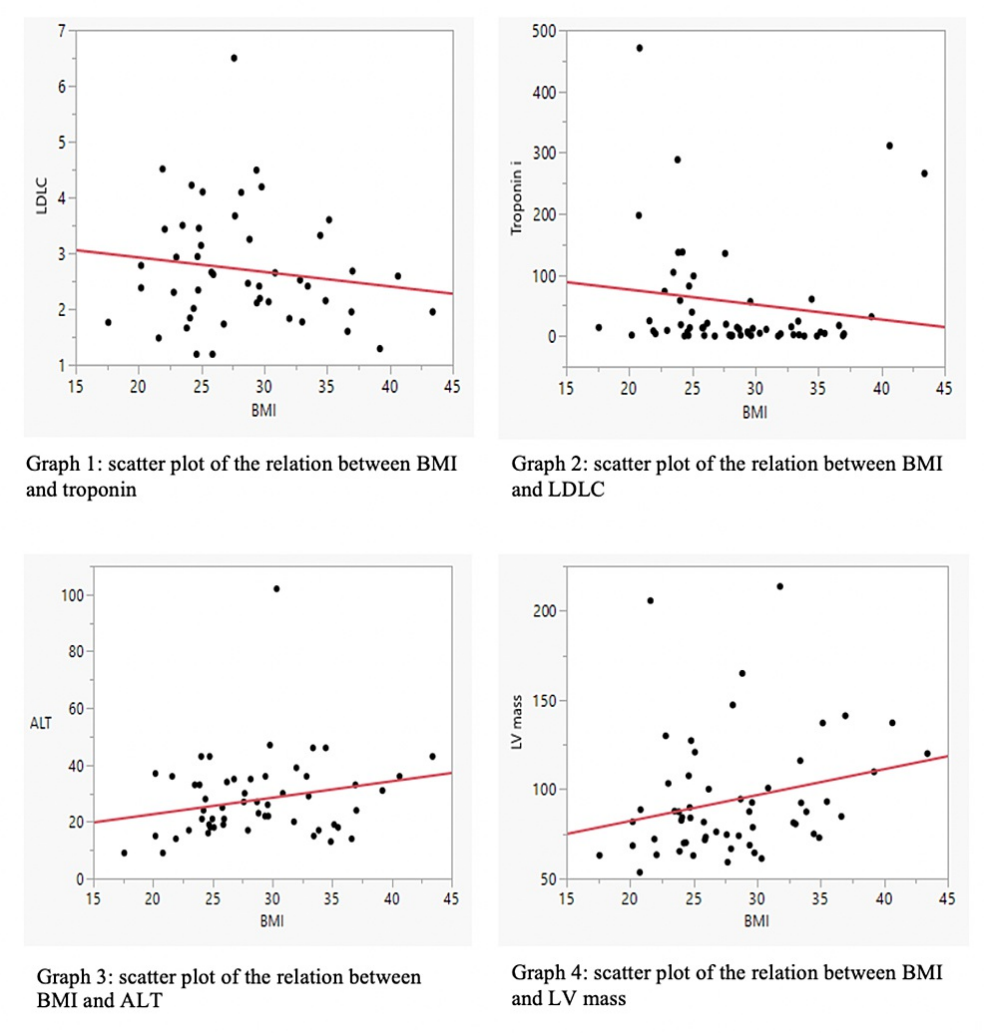
Scatter plots of the relation between BMI and Troponin/LDLC/ALT/LV mass BMI: Body mass index; LDLC: Low-density-lipoprotein cholesterol; ALT: Alanine transaminase; LV mass: Left-ventricular mass

For the laboratory investigation: the mean fasting blood sugar (FBS) was 12.1 ± 5.8. The mean hemoglobin (Hb)A1c was 8.8 ± 1.8. The mean total cholesterol was 4.5 ± 1.1 mmole/L, the mean LDL 2.7 ± 1.1 mmole/L, the mean triglyceride 2.1 ± 1.6 mmole/L, and the mean high-density lipoprotein (HDL) 0.85 ± 0.3 mmole/L. Ten patients (16.4%) had high B-type natriuretic peptide (BNP) levels. The mean hemoglobin was 14.3 ± 1.8 (gm/dL). Nine patients (14.8%) had abnormal creatinine levels. There was no statistically significant association between a high body mass and the total cholesterol, LDL, or aspartate transferase/alanine transaminase (AST/ALT) (Figure 1).

No mortality was recorded in our sample. Patients were divided into two groups: group 1, with BMI<25, and group 2, with BMI≥ 25. Overweight patients had a significantly lower level of troponin and a higher heart rate (Table 1).

**Table 1:** Baseline characteristics and laboratory findings according to BMI group BP: Blood pressure; CK-MB: Creatine kinase-MB enzyme; LDL-C; Low-density lipoprotein cholesterol; AST: Aspartate transferase; ALT: Alanine transaminase; BP: B-type natriuretic peptide; HbA1C: Hemoglobin A1C; BMI: Body mass index

Characteristics	Mean normal weight group (BMI <25) n=23	Mean overweight group (BMI ≥ 25) n=38	P-Value *Significant at 5%
Demographical Characteristics: mean ± SD			
Gender			
M (%)	21(91.3)	30(78.9)	0.294
F (%)	2(8.7)	8(21.1)	
Age in year	56.9 ± 13.2	56.7 ± 10.2	0.923
Clinical parameters			
Heart rate (beat/minute)	77.3 ± 15.9	86.9 ± 18.8	0.045*
Systolic BP (mm.Hg)	133.4 ± 20.5	136.1 ± 22.0	0.640
Diastolic BP (mm.Hg)	82.3 ± 12.6	79.0 ± 12.5	0.319
Level of troponin	105.2 ± 142.3	30.9 ± 67.7	0.004*
CK-MB	123.8 ± 89.4	75.2 ± 63.9	0.068
LDL-C (mmole/L)	2.7 ± 0.9	2.7 ± 1.1	1.000
AST (unit/L)	62.1 ± 80.4	52.7 ± 58.7	0.988
ALT (unit/L)	23.8 ± 10.9	29.9 ± 15.6	0.121
BNP (pg/mL)	459.1 ± 819.8	275.8 ± 607.5	0.894
Hemoglobin (gm/dL)	14.4 ± 1.8	14.3 ± 1.8	0. 770
Fasting blood sugar	10.2±5.2	13.3±5.9	
HbA1C	7.7 ± 2.1	8.3 ± 1.8	0.359
Left Ventricle mass (g)	89.9 ± 31.6	97.2 ± 33.5	0.378
Left Ventricle Ejection Fraction	0.435 ± 0.114	0.456 ± 0.088	0.615

The overweight group was younger, with a higher LV mass, ALT, and HbA1c. The bivariate analysis indicated a fair correlation between the BMI and the LV mass. However, there was a negative correlation between the BMI and the level of troponin (Table 2).

**Table 2:** Correlation between BMI group and laboratory findings MI: myocardial infarction; BP: Blood pressure; CPK-MB: creatine phosphokinase-MB; LDL-C; Low-density lipoprotein cholesterol; AST: Aspartate transferase; ALT: Alanine transaminase; BP: B-type natriuretic peptide; HbA1C: Hemoglobin A1C; BMI: Body mass index

MI measurements	Correlation coefficient	P-Value *Significant at 5%
Age in year	0.042	0.749
Heart rate	0.153	0.239
Systolic BP	0.124	0.343
Diastolic BP	-0.049	0.707
Level of troponin	-0.299	0.019*
CPK MB	-0.228	0.151
LDL-C	-0.102	0.485
AST	0.101	0.439
ALT	0.241	0.074
BNP	0.051	0.776
Hemoglobin	-0.122	0.349
Fasting blood sugar	0.230	0.074*
HbA1C	0.061	0.773
Left Ventricle mass	0.276	0.036*
Left Ventricle Ejection Fraction	-0.021	0.875

There was no significant relationship between the level of troponin and the LV mass, the correlation coefficient was 0.021 and P=0.873

## Discussion

Compared with the normal weight group, the overweight group was noted to be younger age and diagnosed with diabetes mellitus, hyperlipidemia, and more hypertensive particularly at the time of discharge. Obesity can cause insulin resistance, and abnormalities in lipid metabolism (metabolic syndrome), particularly in patients with central adiposity. Obesity induces dyslipidemia, diabetes, hypertension, inflammation, and a pro-coagulant state which are risk factors for the development of CVD, a major cause of mortality related to a high BMI [7]. A significant negative association in infarct size, as evident by the troponin level, was noted in the obese group. To the best of our knowledge, no similar data were reported.

The study may have the potential for cardiovascular prevention, considering that troponin levels might be modified by weight. Weight control could prevent both stroke and MI [8]. In a study, the obese patients demonstrated more impaired LV deformation and adverse LV remodeling, compared with the group who had either normal or overweight. In addition, the normal weight group had the lowest survival despite similar infarct characteristics [9]. We could observe that independent of LV function, there is a potential non-adverse effect of the group with higher BMI when compared to lower BMI. Such observation might point to an alternative pathophysiological process, that leads to a better prognosis in the group with higher BMI in the sitting of ST-elevation myocardial infarction [9].

A study in Japan demonstrated that overweight/obesity was associated with an increased risk of cerebral infarction and hemorrhage in men and women and MI in men [10]. A long-term follow-up study may produce an improved risk assessment as being overweight in the childhood period can cause eccentric left ventricle hypertrophy [11]. Although obese patients had a higher LV mass, it was not statistically significant. An increased body mass has a strong association with left ventricle hypertrophy, compared with hypertension [12]. The obese group had a higher HbA1c level, and a higher BMI had a positive relationship, compared with diabetes, with myocardial dysfunction and cardiomyopathy [13]. Although the obese group in this study had a higher mean LV ejection fraction, a strong relationship was observed between obesity, cardiomyopathy, and heart failure. Normalization of the left ventricular mass in obese and hypertensive individuals requires both normotension and weight loss [14].

Similar observations were reported in Non-ST-elevation myocardial infarction, where patients with a BMI of ≥28 kg/m^2^ had a more adverse outcome and the occurrence of MACE. It was concluded that controlling abdominal obesity might improve the prognosis [15].

The complexity of obesity-related variation in troponin might be part of the spectrum that was noted with other markers linked with high BMI and poor prognosis post-myocardial infarction like asymmetric dimethylarginine (ADMA) and symmetric dimethylarginine (SDMA). The baseline levels and/ or the time course of these two markers are influenced by the BMI [16]. Other markers that demonstrate adverse relation to high BMI in acute myocardial infarction were the high activity of copeptin and MRproADM [17]. On the other hand, myocardial infarction occurring in the underweight group was found to be an independent predictor of sudden cardiac death [18].

Study limitations

The study was limited by the number of recruited patients and the reliance on troponin I. Larger study will emphasize this important risk predictor and the duplication by using troponin T might be an advantage.

## Conclusions

The correlation between myocardial infarction and weight is a complex one. Obese patients who sustained myocardial infarction had a negative correlation with a mean level of troponin. The relatively low troponin in the sitting of obesity needs to be considered when risk stratifying such patients. A higher mean heart rate was also observed. Our study points out that a fair correlation between BMI and LV mass exists.
